# Magnetic Functionalized Nanoparticles for Biomedical, Drug Delivery and Imaging Applications

**DOI:** 10.1186/s11671-019-3019-6

**Published:** 2019-05-30

**Authors:** Simon D. Anderson, Vanessa V. Gwenin, Christopher D. Gwenin

**Affiliations:** School of Natural Sciences, College of Environmental Sciences and Engineering, Bangor University, Bangor, LL57 2UW UK

**Keywords:** Nanoparticle, Drug delivery, Nanoparticle synthesis, Nanomedicine

## Abstract

Medicine is constantly looking for new and improved treatments for diseases, which need to have a high efficacy and be cost-effective, creating a large demand on scientific research to discover such new treatments. One important aspect of any treatment is the ability to be able to target only the illness and not cause harm to another healthy part of the body. For this reason, metallic nanoparticles have been and are currently being extensively researched for their possible medical uses, including medical imaging, antibacterial and antiviral applications. Superparamagnetic metal nanoparticles possess properties that allow them to be directed around the body with a magnetic field or directed to a magnetic implant, which opens up the potential to conjugate various bio-cargos to the nanoparticles that could then be directed for treatment in the body. Here we report on some of the current bio-medical applications of various metal nanoparticles, including single metal nanoparticles, functionalized metal nanoparticles, and core-shell metal nanoparticles using a core of Fe_3_O_4_ as well as synthesis methods of these core-shell nanoparticles.

## Introduction

**Fig. 1:**
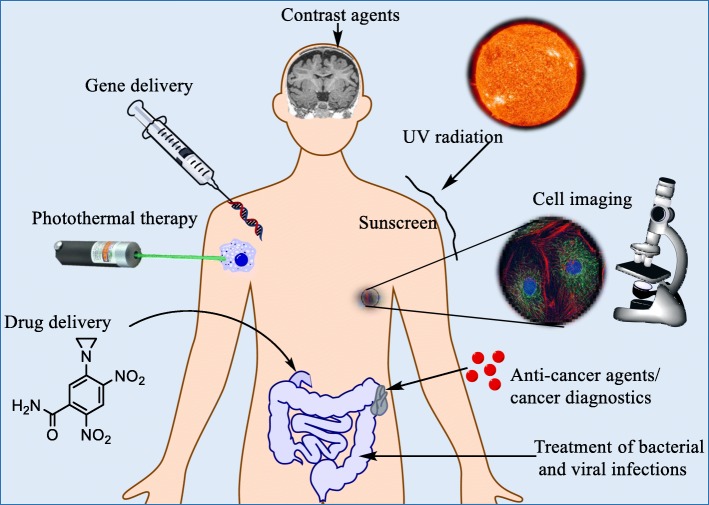
Some of the current uses of nanoparticles in medicine

Metal nanomaterials represent a significant doorway for the future of medicine. Although there is still much unknown about the long-term safety of metal nanoparticles in medicine [1], these particles have already found their place within various biomedical applications such as site-specific imaging in vivo [2–4], cancer detection [5, 6], cancer therapy [7–10], neurodegenerative disease therapy [11–13], HIV/AIDS therapy [14–16], ocular disease therapy [17–19], and respiratory disease therapy [20, 21]. Figure 1 presents a range of current uses for nanoparticles in medicine. Despite the recent advances in nanomedicine, there are still many obstacles in the way of nano-therapy, such as it can be hard to achieve a synthesis route which produces easily repeatable results, with many nanoparticle synthesis methods producing a range in both size [22–24] and shape [25–28] of nanoparticles and/or do not produce the nanomaterials in a large enough quantity to make it economically viable [29]. Another key factor is that it is relatively unknown as to the long-term toxicity of some nanoparticles over a period of time due to how relatively new the field of research is [30–32]. Among the many possible uses of metal nanoparticles lies the area of drug delivery [33, 34]. Due to the large surface area that nanoparticles provide [35], they possess the ability to be able to deliver large quantities of drugs or other medical cargoes [36]. An alternative to single metal nanoparticles is to incorporate a core to the nanoparticle which has alternative properties to the shell material, and one example of this is to incorporate a magnetic core. One challenge that still presents itself is synthesizing core-shell nanoparticles; there are many ways to synthesize nanoparticles [37], but new challenges emerge when attempting to synthesize a core-shell nanoparticle [38].

This review first focuses on some of the methods currently used to generate core-shell nanoparticles focusing on using cores of Fe_3_O_4_ and coatings of gold or silver. We then examine current bio-medical applications of single metal nanoparticles, their limitations, and how to overcome them with the application of magnetic cores.

## Synthesis of Core-Shell Nanoparticles

Methods for the synthesis of metallic nanoparticles have been known for many years, for example, Stevenson et al. published a synthesis for gold nanoparticles via the reduction of HAuCl_4_ in 1951 [39]. Since then, there have been many different routes for nanoparticle synthesis such as gas deposition [40], sol-gel [41], and aerosol/vapor phase [42]. However, a new challenge presents itself when attempting to synthesize metal nanoparticles consisting of a core-shell structure, in which one metal forms the core and a second metal forms the shell, for example Fe particles degrade in water, whilst HAuCl_4_ is a strong oxidizing agent [38]. One such example that will be discussed further is using a Fe_3_O_4_ (iron oxide) core and gold as the coating shell. In the preparation of such core-shell metal nanoparticles, two of the biggest issues are attempting to control the rate of coating and controlling the uniformity of the coating to create a solution of nanoparticles which are all of very similar shape and size [43]. Coating of gold or silver onto an iron oxide core can be divided into two main categories: direct coating of gold/silver onto iron [44] or using an intermediary layer to act as a glue between the gold and the iron layer [45]. The former category will be discussed here. The following text describes some methods that have been devised to synthesize gold- and silver-coated Fe_3_O_4_ nanoparticles.

### Reverse Micelle Synthesis

A popular route for synthesizing metal nanoparticles is to use the reverse micelle method, or sometimes called the microemulsion route [46]. This method was first introduced in the 1980s when colloidal solutions of rhodium, platinum, and palladium nanoparticles were first synthesized [47].

Micelles are formed when molecules with hydrophobic and hydrophilic constituent parts come into contact with either an aqueous or hydrophobic phase [48]. The micelles will organize themselves in such a way that allows the hydrophilic part to be in contact with the aqueous phase and the hydrophobic constituent facing the hydrophobic phase [49]. In essence, a spheroid is formed with an inner shielded phase, which can furthermore contain a cargo [43, 50–52].

There are different approaches to the microemulsion route and these include water-in-oil (w/o) [53] and water-in-supercritical CO_2_ (w/sc-CO_2_) [54]. A w/o emulsion occurs when water is dispersed in a hydrocarbon-based continuous phase [53], thermodynamically driven surfactant self-assembly, and then generates the reverse micelles, with spherical micelles being the most common shape [43]. Any added polar or ionic materials added to this mixture become compartmentalized within the micelles, and nanoparticles are then formed when the micelle membranes come into contract with each other through Brownian motion [55]. A w/sc-CO_2_ emulsion involves using a fluid (CO_2_) that is in a supercritical state, i.e., above both its critical pressure and temperature [56]. This method holds particular interest as it is a more “green” approach to nanoparticle synthesis as no toxic organic solvents are required. It is also easier to recoup the product by simply lowering the pressure and releasing the fluid as CO_2_ gas [57].

**Fig. 2:**
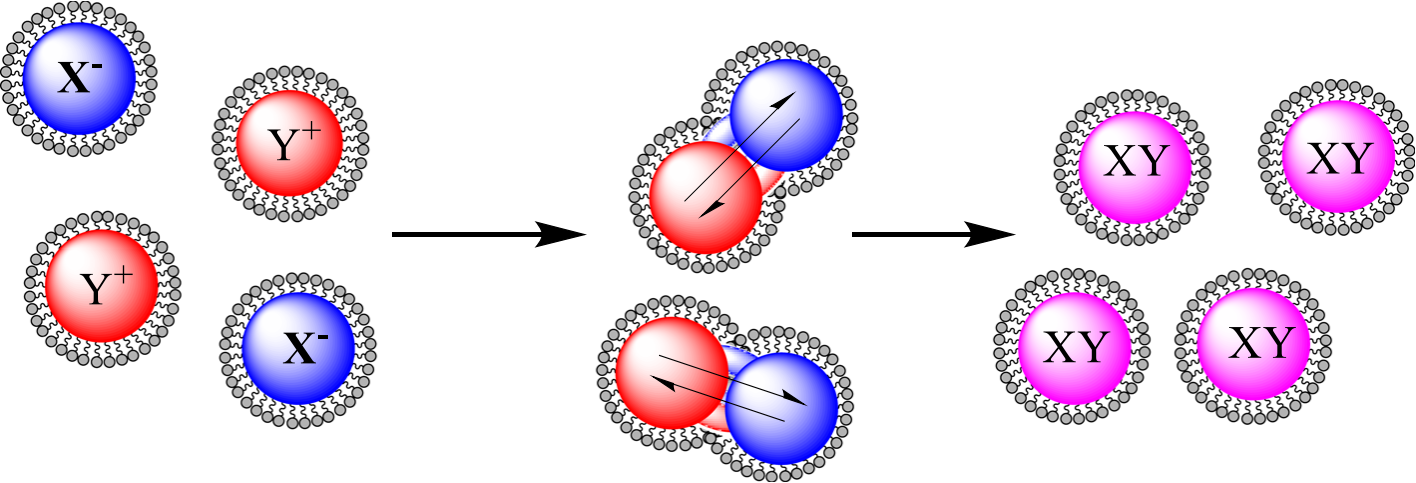
A generic representation of the interaction of reverse micelles containing salts the react to form metal nanoparticles

The reverse micelle route has been adapted from synthesizing metal nanoparticles to coating previously synthesized nanoparticles [58]. The first gold-coated iron oxide (Au-Fe_3_O_4_) nanoparticles synthesized in reverse micelles were done so almost 20 years ago [59]. This synthesis of Au-Fe_3_O_4_ nanoparticles was done using a H_2_O/CTAB (cetyltrimethyl ammoniumbromide) system to produce the micelles with sodium borohydride (NaBH_4_) as the reducing agent, reducing gold chloride (HAuCl_4_) onto the iron core. This synthesis produced a nanoparticle dispersion with an average size of 12 nm. Since this is the first production of Au-Fe_3_O_4_ NPs using microemulsions, there has been a range of Au-Fe_3_O_4_ NPs synthesis routes discovered [46, 60–63]. Figure 2 is a generic representation of how the nanoparticles are formed using the reverse micelle route.

Lin et al. published a slightly modified method to coat Fe_3_O_4_ with gold using a reverse micelle method [60]. The synthesis also employs a system using CTAB as the surfactant to form the reverse micelle, but with 1-butanol as a co-surfactant and octane as the oil phase, adding a water solution containing the metal ions using NaBH_4_ to reduce HAuCl_4_ onto the surface of the iron oxide nanoparticles. The reported optical results of the coated particles showed a shift in the absorbance peak of the UV/vis spectra from the gold colloid (526 nm) to the Au-Fe_3_O_4_ (555 nm). The TEM results of the coated particles indicated a size distribution of 5–15 nm, with an average size of 10 nm. This method was repeated by Pana et al. with a slightly larger size distribution of 5–35-nm-sized Au-Fe_3_O_4_ nanoparticles [63]. In addition, a very similar system has been employed by Seip et al. with the exception of using hydrazine to reduce the HAuCl_4_ [64].

The coating of Fe_3_O_4_ nanoparticles is not limited to just gold; Lopez Perez et al. reported on the synthesis of iron oxide nanoparticles using a system containing cyclohexane/Brij-97 (co-surfactant) and an aqueous phase with iron salts of FeSO_4_.7H_2_O and FeCl_3_.6H_2_O [65]. This system has been coated with both silver [58] and gold [46], producing 13-nm particles. An alternative method is reported by Tamer et al. for the synthesis of Au-Fe_3_O_4_ nanoparticles [62]. This method employs a co-precipitation of iron salts in NaOH, which were then washed in HClO_4_ to produce oxidized Fe_3_O_4_ nanoparticles. Coating of gold onto the Fe_3_O_4_ NPs occurred via the reduction of HAuCl_4_ by NaOH delivered to the system by CTAB micelles. Au-Fe_3_O_4_ NPs were produced with an average size of 23.5 nm. After characterization, particles were then modified with various functional groups to form a self-assembled monolayer (SAM) and further used for the capturing and detection of *Escherichia coli*.

A modified version of the reverse micelle synthesis has been done by Zhang et al. involving the use of a laser as the initiator for the coating of iron nanoparticles with gold [66]. The process involves making a reaction mixture of iron nanoparticles encapsulated in CTAB micelles, gold nanopowder in water, and octane, then irradiating with a pulsed laser while vigorously stirring the reaction. The laser irradiation facilitates the thermal decomposition of the gold nanoparticles. Gold atoms and clusters formed around the iron nanoparticles, forming gold-coated iron nanoparticles. The TEM results for the Au-Fe nanoparticles synthesized this way gave an average size of 18 nm with a size distribution of ±36 nm.

### Thermal Synthesis

Among the various methods of gold shell-iron core nanoparticle synthesis lies a thermal route, wherein the reaction involves heating the reaction mixture to above its boiling point [67], and sometimes refluxing [68, 69]. There are two main categories for this type of synthesis: hydrothermal (water-based solvent) [70, 71] and solvothermal (organic-based solvent) [68, 72]. While there are many techniques for synthesizing metal nanoparticles via the thermal route [73–78], it is not possible to achieve the synthesis of the cores and coating of gold in a one pot reaction [68, 69, 72, 74, 77, 79–81], and in some cases, Fe_3_O_4_ cores are synthesized via a reverse micelle route [70] or a colloidal route [78] and then the particles are coated using a hydro- or solvothermal technique [70, 76, 78]. While there are a variety of solvent systems that are used in these synthetic methods, the majority of routes involve the addition of either iron oxide nanoparticles to boiling HAuCl_4_ or the inverse of HAuCl_4_ being added to boiling solutions of iron oxide nanoparticles [74, 79].

**Fig. 3:**
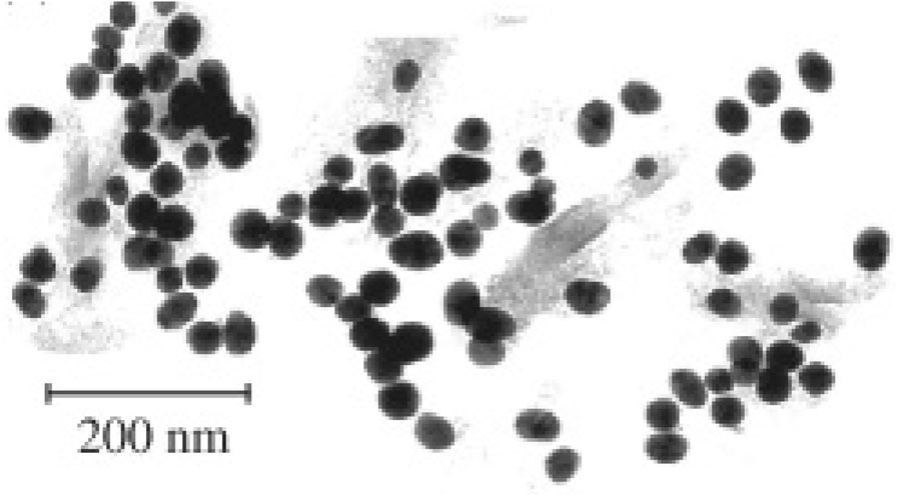
A TEM image of the nanoparticles synthesized by Rudakovskaya et al. As can be seen, the nanoparticles are roughly spherically shaped with an average size of 30 nm [76]

A method for the synthesis of Au-Fe_3_O_4_ nanoparticles has been done by Rudakovskaya et al. via a hydrothermal technique [76]. The principle of the method follows the addition of Fe_3_O_4_ nanoparticles to a boiling HAuCl_4_ solution. TEM analysis of these nanoparticles indicated an average size of 30 nm, with a general spherical shape and a size distribution between 20 and 35 nm; these images can be seen in Fig. 3.

### Colloidal Synthesis

Colloidal synthesis techniques offer a simple yet effective way of synthesizing metal nanoparticles [82]. Colloidal techniques often offer a level of simplicity over other techniques for nanoparticle synthesis, without the need for different solvents, or that it can be carried out at room temperature [83, 84]. The basic principles of the synthesis involve dispersing different metal ions in an aqueous phase, adding a reducing agent to the mixture, then mixing at a controlled temperature to form insoluble nanoparticles [39]. Colloidal synthesis routes offer the benefit of not having to involve potential toxic solvents in the synthesis (ideal if the nanoparticles are intended for biological use). However, there are some limitations to colloidal routes such as it can be hard to control the size distribution of the final synthesized nanoparticles [85] and the shape of the nanoparticles can be heavily influenced by reagent concentration [85]. On the positive side, it can however be easier to produce nanoparticles in a larger quantity [86]. This method for metal nanoparticle synthesis has been around for many years, being used for the synthesis of different types of nanoparticles such as silver [87] and gold [39, 88].

**Fig. 4:**
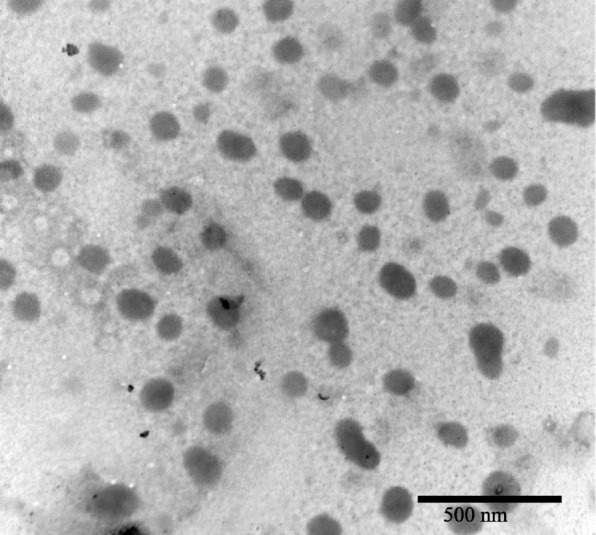
A TEM image of the nanoparticles synthesized by Rawal et al. These nanoparticles have a size distribution of 20–100 nm [83]

This basic method has been advanced and developed to produce different synthetic routes for the formation of gold-coated iron oxide nanoparticles [83, 84, 89–97]. Most of the methods for the synthesis of gold-coated iron oxide revolve around using various reducing agents to reduce HAuCl_4_ onto the surface of the iron oxide. Nadagouda et al. offer a proposed “green” synthetic route, using ascorbic acid to reduce HAuCl_4_ [84]_._ This method however seems to show little to no control over size or shape of the coated nanoparticles due to the lack of capping agent (an agent that binds to the outside of the nanoparticle that stops further “growth” of the nanoparticle) used in the synthesis [98]. A method which does show more control over the shape and size of synthesized coated particles is presented by Pal et al. [95] This method employs gold acetate as the gold salt, which is reduced onto the surface of 6-nm Fe_3_O_4_ nanoparticles to create 7-nm-sized Au–Fe_3_O_4_ particles, which are spherical in shape. A rapid method for coating Fe_3_O_4_ nanoparticles is presented by Rawal et al. which involves dispersing Fe_3_O_4_ nanoparticles in a solution of HAuCl_4_, then mixing with ethanol [83]. After 15 min at room temperature, the reaction was stopped and the Au-Fe_3_O_4_ nanoparticles were then separated with a magnet. TEM analysis of the purified solution showed that the particles produced ranged in size from 30 to 100 nm and had varied shapes across the sample; these images can be seen in Fig. 4. While this synthesis technique produced the coated nanoparticles quickly, it does not appear to be a very efficient synthesis for production of uniformly shaped and sized particles [83].

While some techniques offer just the reduction of gold salts, others prefer to put the reducing agent onto the surface of the iron, such as hydroxylamine [90, 93]. In many cases when Fe_3_O_4_ nanoparticles are coated with gold, the reduction of a gold salt yields standard gold nanoparticles as well [74], so the addition of the reducing agent onto the surface of the iron nanoparticles aims to improve the efficiency of the coating and is intended to lower the quantity of gold nanoparticles produced as a by-product [93].

Another technique involves seeding gold onto the surface of magnetic nanoparticles which provides a more direct route of getting gold to nucleate around the magnetic core of the nanoparticles [91, 92, 97]. This technique involves binding gold seeds, which are smaller than the iron oxide nanoparticles in solution, to the surface of the iron oxide. When the HAuCl_4_ is reduced in solution, the Au^+^ ions will seed onto the iron oxide and form a shell around the iron oxide nanoparticles. This gold seeding has been successfully employed by several groups; Goon et al. used polyethyleneimine to control the seeding of gold onto the surface of Fe_3_O_4_, producing fully coated nanoparticles. [91] However, the synthesized Au-Fe_3_O_4_ particles displayed high polydispersity, with particle size ranging from 40 to 110 nm. Levin et al. managed to produce gold shell-magnetic core nanoparticles with a size range of 50–70 nm, using a core functionalized with organosilane molecules to bind to the gold seeds [92]. Seeding of gold nanoparticles onto an iron core can be demonstrated with a variety of core shapes, for example, Wang et al. demonstrated gold seeding onto rice-shaped “Nano rice” Fe_3_O_4_ structures, which then led to a complete thick gold shell when gold was reduced onto the surface [97].

## Bio-Medical Applications of Metal Nanoparticles

### Antimicrobial Agents

Bacterial infections are very common, with antibiotics being a primary method of treatment since the discovery of penicillin in 1928 by Alexander Fleming [99]. Nanomedicine provides us with a new, broad range of possible treatment modalities, with metal nanoparticles being explored for future treatments [100]. Table 1 lists some of the nanoparticles that have been explored for antimicrobial applications. One material that has been examined for its potential use is silver, which has shown to have a variety of biomedical uses [101], for example, Sreekumar et al. utilized silver nanoparticles as part of a network of antimicrobial fibers. The nanoparticles varied in size from 20 to 120 nm, with an antibacterial efficacy against *Escherichia coli* as high as 94.3% compared to the fibers without silver nanoparticles [102]. While it has been shown that an antibiotic such as ampicillin is capable at achieving a kill rate of ≤ 99.9% in *E. coli* [103], the same study also reported the emergence of resistance to ampicillin in certain strains of *E. coli*. On this same note, it has been reported that *E. coli* can develop a resistance to silver nanoparticles; however, this resistance is not a genetic change, but it is a physical response that attempts to cause the colloidal nanoparticles to aggregate [104]. Also employing silver for its antibacterial properties, Holtz et al. designed a system of 60-nm silver vanadate nanowires ‘decorated’ with silver nanoparticles with a diameter of 1–20 nm [105]. This system showed to be promising against three *Staphylococcus aureus* strains and also interestingly had a much lower growth inhibiting concentration against methicillin-resistant *Staphylococcus aureus* (MRSA) than the antibiotic oxacillin.

**Table 1:** List of antibacterial properties that have been exhibited by some metal nanoparticles and metal nanoparticle conjugates

Type of nanoparticle	Size (nm)	Antimicrobial application	Mechanism of action	Ref
Silver as part of network of fibers	20–120	*E. coli*	Bacterial growth inhibition	[102]
Silver vanadate nanowires	1–20	*S. aureus*	Bacterial growth inhibition	[105]
Naked silver	10–25	*C. albicans*, *P. fluorescens*, *E. coli*	Bacterial growth inhibition	[106]
Thioguanine-capped gold	3–4	*E. coli*, *A. fumigatus*, *P. aeruginosa*, and anticancer effect against Hep2	Bacterial growth inhibition, cellular toxicity	[107]
Naked gold	25	*C. pseudotuberculosis*	Vacuole formation in cell wall and agglomeration of NPs within cells	[108]
Naked gold	6–40	*S. aureus*, *K. pneumonia*, *B. subtilis*	Bacterial growth inhibition	[109]

A silver nanoparticle synthesis was reported by Verma et al. where they employed their nanoparticles against the bacteria: *Pseudomonas fluorescens*, *E. coli*, and the fungus *Candida albicans* [106]. The silver nanoparticles had an average minimum inhibitory growth concentration of 5.83 μg/ml across the three strains, compared to some commonly used antibiotics such as ampicillin and neomycin which have minimum inhibitory growth concentrations of 4.0 μg/ml and 16.0 μg/ml, respectively, against strains of *E. coli* [110]. Of potential interest is the properties the nanoparticles displayed against *P. fluorescens* an *C. albicans*, both of which are associated with causing disease in immunocompromised patients [111]. Further investigations might find that the silver nanoparticles are a more efficient way to treat the pathogens than some of the most commonly used antibiotics, such as amphotericin B, which has extensive side effects [112].

The synthesis of thioguanine-capped gold nanoparticles has been reported by Selvaraj et al. where an enhanced antimicrobial effect against several bacterium, including: *E. coli*, *Aspergillus fumigatus*, and *Pseudomonas aeruginosa* [107]. It was found that the thioguanine-capped gold nanoparticles were more effective than unconjugated thioguanine as anticancer and antimicrobial agents, with their activities showing potential use as carriers for cancer drugs. In a similar manner, gold nanoparticles have been reported to have an antimicrobial effect on *Corynebacterium pseudotuberculosis* [108], nanoparticles with an average size of 25 nm, using a dose of 50 μg/ml showed a bacterial growth inhibition of 95% after 20 min of exposure. Similarly, naked gold nanoparticles were shown to have an antimicrobial effect on a variety of gram negative and gram positive bacteria including *S. aureus*, *Klebsiella pneumonia*, and *Bacillus subtilis* [109]. A dose of 1.35 μg/ml of AuNPs showed a growth inhibition of 46.4±0.4%, 38.3±0.2%, and 57.8±0.2% for *S. aureus*, *K. pneumonia*, and *B. subtilis*, respectively.

### Antiviral

As with antibacterial applications, metal nanoparticles have shown to be promising in antiviral applications; Table 2 demonstrates a range of nanoparticles that have been shown to possess antiviral properties and could potentially be applied when treating viruses. Both naked and coated silver nanoparticles [113–116] have been shown to have a range of antiviral applications when in the nano-scale range.

**Table 2:** Some of the metal nanoparticles and metal nanoparticle conjugates that have been demonstrated as having antiviral properties

Type of nanoparticle	Size (nm)	Antiviral application	Mechanism of action	Ref
AgNPs	10–50	Hepatitis B virus (HBV)	Interaction with DNA and/or binding with virus particles	[113]
Ag-PS-NPs	10–80	Monkeypox virus (MPV)	Blocking of virus-host cell binding and penetration	[114]
PVP-AgNPs	30–50	Human immunodeficiency virus type 1 (HIV-1)	Prevention of HIV-1 transfection	[115, 116]
Au-MES	4	Herpes simplex virus type 1 (HSV-1)	Competition with host cell binding	[117]
Gold coated with an amphiphilic sulfate ligand	2	Human immunodeficiency virus type 1 (HIV-1)	Binding to gp120	[118]
Copper iodide (CuI) nanoparticles	100–400	Feline calicivirus (FCV)	ROS generation and subsequent capsid protein oxidation	[119]
Copper iodide (CuI) nanoparticles	160	Influenza A of swine origin (H1N1)	Generation of hydroxyl radicals and degradation of viral proteins	[120]

Hepatitis B (HBV) is a viral infection that currently affects 257 million people around the world and was responsible for 887,000 deaths in 2015 according to the World Health Organization [121]. Small (10–50 nm) naked silver nanoparticles have been tested as a possible treatment for HBV [113] and were shown to bind efficiently to HBV and further inhibit the production of HBV RNA. The mode of action is hypothesized to be due to the AgNPs binding to the HBV dsDNA (double-stranded DNA). Rogers et al. have demonstrated a use for silver nanoparticles, both naked and with a polysaccharide coating as an antiviral agent against monkeypox virus (MPV) [114]. The nanoparticles were tested in vitro against MPV at a range of concentrations between 12.5–100 μg/ml; the results of the study showed that all of the concentrations of polysaccharide-coated silver nanoparticles (Ag-PS-NPs) used were able to reduce MPV-induced plaque formations in vitro.

Silver nanoparticles may even have a role to play in the treatment of human immunodeficiency virus (HIV) [115, 116]. HIV is a major health concern, with WHO estimating that 36.7 million people are living with HIV as of 2016 [122]. It is important that treatments for HIV are discovered and implemented quickly and efficiently; Lara et al. have demonstrated the effect of silver nanoparticles (30–50 nm) on HIV-1 isolates showing inhibition of all strains of HIV-1 isolates [116]. The naked nanoparticles showed an overall IC_50_ of 0.44 mg/ml ± 0.3 against HIV-1, with the mechanism of viral inhibition shown to be the inhibition of virus-host cell binding, specifically the silver nanoparticles inhibit the interaction between the gp120 protein (an envelope glycoprotein) and the target cell membrane receptors. Also demonstrated by the same group was the ability for silver nanoparticles coated with polyvinylpyrrolidone (PVP) to prevent the transfection of HIV-1 into a human cervical tissue explant model [115]. Specifically, 0.15 mg/ml PVP-coated silver nanoparticles (PVP-AgNPs) inhibited infection by HIV-_IIIB_ and HIV-_AZT-RV_ isolates. This concentration of PVP-AgNPs also induced a proliferation of lymphocytes (immune cells) to the site of infection, in comparison to the control sample [115].

It is not only silver and coated silver nanoparticles that have been employed against viruses: 2-nm gold nanoparticles coated with an amphiphilic sulfate ligand were also shown to be effective against HIV-1 [118]. These particles were shown to target the fusion process of the virus and were shown in vitro to bind to gp120 protein and directly neutralize the HIV-1 infection. Mercaptoethanesulfonate-coated gold nanoparticles (Au-MES) showed an inhibition of herpes simplex virus type 1 (HSV-1) infection, possibly by inhibiting the virus binding to the host cell, cell to cell viral spreading, or alteration of cell susceptibility to viral infection induced by the presence of the nanoparticles [117].

Copper-iodide nanoparticles (CuI-NPs) have been shown to have antiviral properties on several different viruses: feline calicivirus (FCV) [119] and more interestingly influenza A virus of swine origin (H1N1) [120]. One hundred to 400 nm CuI-NPs showed an antiviral property when utilized against FCV, and it was hypothesized that monovalent Cu ions were responsible for the production of a reactive oxygen species (ROS) that caused subsequent capsid protein oxidation, leading to FCV inactivation. H1N1 virus was also shown to be inhibited by CuI-NPs, in a very similar manner, namely the production of hydroxyl radicals, leading to protein degradation. However, these radicals might also prove to be toxic to non-infected tissues, which would be important to determine before a treatment would be approved for use [123].

### Imaging

**Fig. 5:**
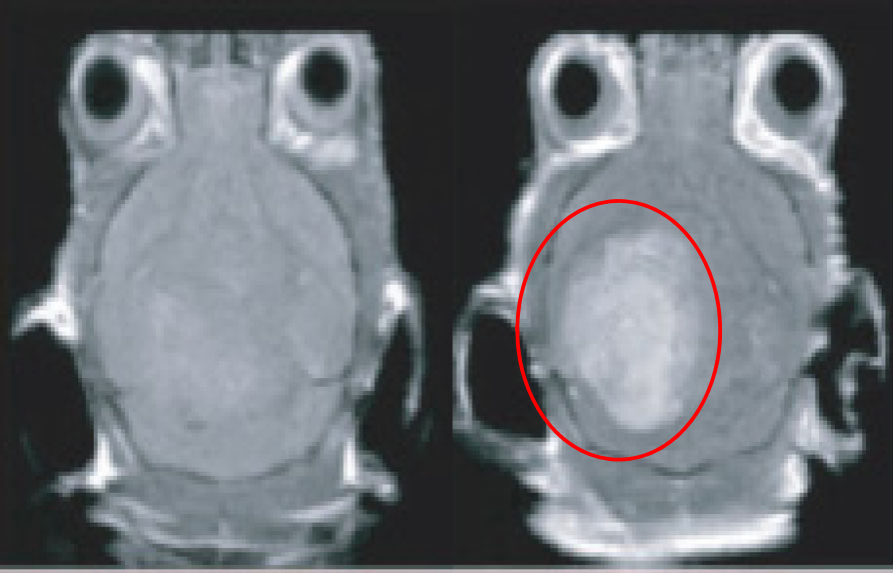
An MRI contrast image of a rat cerebral cortex pre- (left) and post-treatment (right). The area containing the AuNPs is ringed in red

Magnetic resonance imaging (MRI) scanning is a very useful tool for medical diagnosis and provides clear anatomical images. Using MRI, one can visualize the blood flow, physiochemical traits, and the states of tissues and organs in the body [124]. Contrast agents are often employed in MRI for improved diagnostic sensitivity [125]. Conventionally used contrast agents are chelate-based, but the major drawbacks of current contrast agents are their biological stability and their toxicity levels when accumulated in cells [126]. For example, some contrast agents are iodine-based, and it has been reported that iodinated contrast media exposure is associated with subsequent development of incident hyperthyroidism and incident overt hypothyroidism [127]. Alternatives have been developed to provide an improved scanning efficacy by reducing the negative impact contrast agents can have on the body [128]. Alternatives include metal nanoparticles possibly conjugated with an agent which acts in a similar manner to a contrast agent for MRI scanning [129]. Figure 5 is an MRI contrast image of a rat cerebral cortex pre- and post-treatment of AuNPs [130].

Table 3 shows some of the nanoparticles that have been explored for use in medical imaging. Some computed tomography (CT) contrast agents have issues including short circulation half-lives [131] and potential tissue damage [130]. Due to this, metal nanoparticles have also been investigated for use in CT imaging [132]; Au nanoparticles show promising use in imaging due to their X-ray attenuation [133]. Kojima et al. showed that gold nanoparticles conjugated with a PEGylated dendrimer (PEG-AuNPs) made for a superior contrast agent in vitro *as well as* for X-ray computed tomography, compared to the commercially available iodine agent iopamidal [134]. The PEG-AuNPs showed a higher contrast efficiency than the commercially available iopamidal, with rapid excretion from the body [135]. The authors also noted that the PEG-AuNPs had photocytotoxic properties to enable photothermal therapy.

**Table 3:** Some examples of metal nanoparticles and metal nanoparticle-conjugates that have been investigated for their use in medical imaging

Type of nanoparticle	Size (nm)	Scanning type	Ref
PEG-AuNPs	3–8	CT	[135]
Modified AuNPs	17–23	SPECT/CT	[136]
AuNPs	130–147	PA	[137]
AuNPs with citraconic amide moieties	10	PA	[138]
AuNPs in combination with radiotherapy	25	Dual-energy CT	[139]

Li et al. have demonstrated the use of coated AuNPs as an imaging tool for atherosclerosis; the AuNPs were applied in a type of medical imaging called “single-photon emission computed tomography” (SPECT) [136]. This type of imaging is very similar to using a gamma camera, but it is able to provide true 3D images that can be sliced, rotated, and manipulated to achieve a more accurate analytical technique [136]. The modified nanoparticles specifically targeted atherosclerosis plaques containing apoptotic macrophages, indicating a useful tool for invasively accurate detection of atherosclerosis plaques [136].

**Fig. 6:**
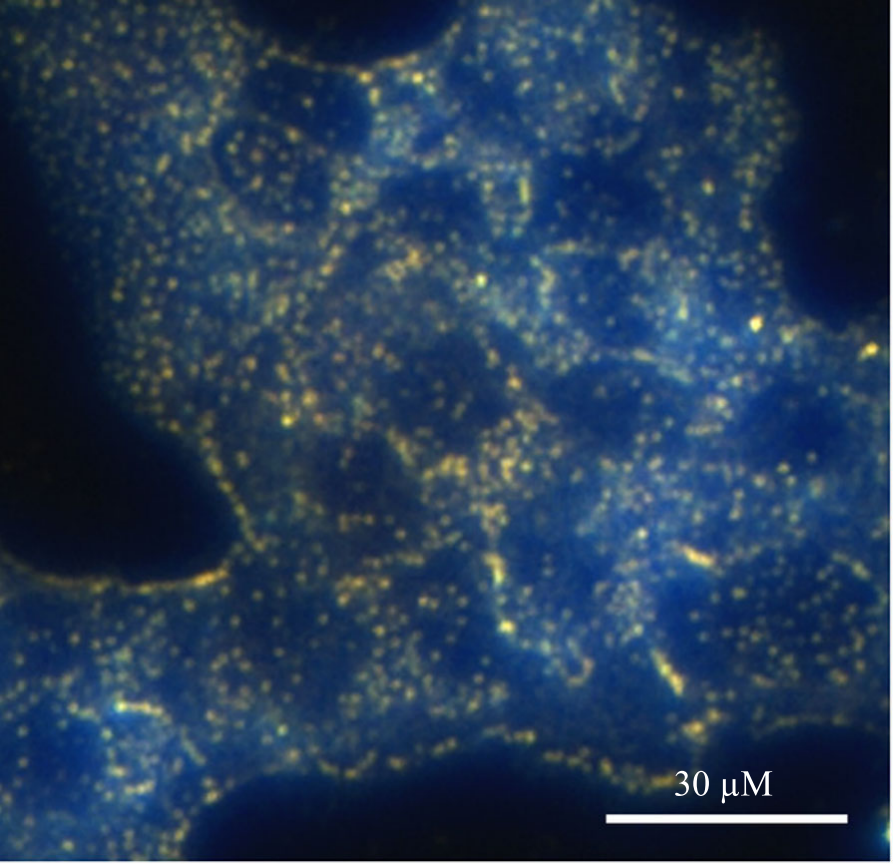
Darkfield imaging of A431 lung cancer cells treated with AuNPs; the bright yellow/orange dots are nanoparticles within the cells

AuNPs have previously been demonstrated to be a possible agent for photoacoustic imaging (PA), showing high spatial resolution and sensitivity [137]. PA relies on the detection of ultrasonic waves which are emitted from tissues when exposed to non-ionizing pulsed laser irradiation [140]. The intensity/magnitude of the ultrasonic emission is responsible for the image contrast, therefore any agent that can both absorb the laser pulses and then give off heat as a result will increase the magnitude of the ultrasonic emission and AuNPs possess the ability to do both of these [141, 142]. AuNPs are potentially better than organic dyes due to the organic dyes’ susceptibility to photo-bleaching and rapid clearing from the blood [143]. AuNPs also have use in cell imaging for examining movement of nanoparticles within cells when conjugated with various cargoes. Figure 6 is a darkfield imaging of A431 lung cancer cells treated with AuNPs that target epidermal growth factor receptor, and the bright areas within the cells are the nanoparticles indicating their locations within the cells [144].

### Biomedical Cargo Delivery

Nanoparticles make for an ideal molecule for drug delivery due to the huge surface area to the volume ratio they provide when compared to their bulk material [145]. In addition, it is possible to engineer nanoparticles to either avoid or interact with the immune system in specific ways [146, 147]. For example, it has been demonstrated that an increased hydrophobicity of nanoparticles/sub-groups conjugated to the nanoparticles illicit and increased immune response by measuring cytokine mRNA levels in mice [146]. Focusing in the opposite direction, it has been suggested that nanoparticles can be conjugated with various ligands to directly activate the immune system to target the destruction of a tumor [148] or by accumulation in the liver or spleen for the generation of tolerance or immunity, respectively [147].

Gold nanoparticles have been extensively studied for their delivery of medical cargo, for example, Bhumkar et al. have explored the application of AuNPs for trans-mucosal delivery of insulin. Gold nanoparticles were synthesized in the presence of chitosan, which acts as a polymeric stabilizer [149]. These nanoparticles were then loaded with insulin and administered both nasally and orally to diabetic rats. The results showed an overall reduction in the rat’s blood glucose levels, an indication of successful movement of the nanoparticles through the mucosal membranes and into the bloodstream.

More recently “smart” AuNPs have been employed in PA [138]. These nanoparticles are roughly 10 nm in diameter and are functionalized with citraconic amide moieties which are susceptible to hydrolysis. The citraconic amides are converted into positively charged primary amino acids at a mildly acidic pH, while the surface molecules adopt negative charges at physiological pH [138]. Combined, these two properties cause the “smart” nanoparticles to adopt both positive and negative charges allowing them to aggregate rapidly due to electrostatic attraction. These nanoparticles are referred to as “smart” due to the nanoparticles presenting cancer-specific properties and accumulate rapidly and efficiently in cancer tissues and show a much lower accumulation in normal tissues [150].

Gold nanoparticles can also be used as a delivery system for nucleic acids [153], including oligonucleotides [151] and small interfering RNA (siRNA) [154]. Many different methods have been developed to functionalize AuNPs with nucleic acids, for example, Yonezawa et al. have synthesized gold nanoparticles modified with thiocholine, which then bound to DNA and formed a fusion of wire-like structures throughout the DNA [155]. Sandström et al. demonstrated the ability to bind nucleic acids onto gold nanoparticles [151], and a similar modification has been done by Rosi et al. where tetrathiol-modified antisense oligonucleotides were bound to 13-nm gold nanoparticles [152]. Being able to conjugate nucleic acids to nanoparticles opens up the possibility of targeted gene delivery, which could, for example, lead to genes coding for a specific protein to be delivered to a cell that was either deficient in that protein or could not produce the protein themselves [156]. It has also been exhibited that gold nanoparticles modified with DNA can transfect cancer cells [157] (Table 4).

**Table 4:** A range of nanoparticle conjugates that have been examined for medical delivery of cargos

Type of Nanoparticle	Size (nm)	Medical delivery application	Ref
Chitosan stabilized AuNPs	10–50	Delivery of insulin across transmucosal membranes	[149]
AuNPs conjugated to an oligonucleotide modified with thiol groups	10–20	Delivery of nucleic acids as a potential for gene therapy	[151]
AuNPs conjugated to antisense oligonucleotide modified with tetra-thiol groups	13	Delivery of nucleic acids as a potential for gene therapy	[152]

### Anticancer Drug Delivery

Cancer is one of the world’s leading killers with large areas of scientific research being dedicated to the fight against cancer, and nanoparticles offer a new doorway into methods to target and treat cancer. Table 5 presents a selection of nanoparticle/drug conjugates that have been tested for anticancer treatments. Paciotti et al. have investigated the application of PEGylated AuNPs as a carrier for tumor necrosis factor (TNF) which is a cell-signaling protein that possess the ability to induce apoptosis in healthy cells [158]. The Au-PEG-TNF nanoparticles were injected intravenously and agglomerated significantly more in MC-38 colon carcinoma cells compared to other healthy cells/tissues. The TNF not only gave therapeutic action on the MC-38 cells, but also seemed to possess a targeting property, indicated by the lack of agglomeration in healthy cells. Another interesting observation reported was the ability for the Au-PEG-TNF nanoparticles to diminish a tumor mass compared to “free” TNF.

**Table 5:** A range of nanoparticle conjugates that have been examined for anticancer therapy

Type of nanoparticle	Size (nm)	Medical delivery application	Ref
PEGylated AuNPs conjugated with TNF	30–34	Delivery of TNF to cancer cells targeted by the TNF itself, TNF induces cell apoptosis	[158]
AuNPs conjugated with folic acid using a PEG linker	10	Delivery of folic acid (vitamin B9), a precursor for nucleic acid production	[159]
AuNPs loaded with doxorubicin	30–40	Delivery of doxorubicin-loaded gold nanoparticles for tumor targeting/therapy	[160]
AuNPs coated with a tumor specific uptake peptide	25–40	Drug delivery to lymphoma cells with gold nanoparticles conjugated with cellular uptake peptides specific to lymphoma cells	[161]

Doxorubicin is a widely used cancer therapeutic agent but has dose-limiting associations with cardiotoxicity. A gold nanoparticle-doxorubicin conjugate has been developed that demonstrates little no to cardiotoxicity to mice while being able to treat cancer [160]. Dixit et al. demonstrated the selective delivery of folic acid-coated AuNPs into folate receptor (FR) positive cancer cells, whereas when compared with a cell line that did not have folate receptors, uptake was shown to be minimal [159]. These results demonstrated the use of folate to target metal nanoparticles to FR-positive cancer cells for tumor imaging and ablation.

## Limitations of Single Metal Nanoparticles and Overcoming Them

The principal obstacle with nanoparticle drug delivery is the ability to direct the nanoparticle to the target area [162, 163]. There are several methods in use for metal nanoparticle targeting such as antibodies [164–166] and homing peptides [167, 168]. There are however limitations to these methods, with the biggest being that before they even reach the desired target cells they have to pass through a variety of other barriers, such as blood vessels and the blood-brain barrier [169]. One way to overcome this targeting limitation is to use magnetic nanoparticles [170]. A magnetic nanoparticle-targeting system works by directing the nanoparticles to a target site using an external magnetic field, it has already been demonstrated that the magnetic anisotropy of the nanoparticle is a very important factor for medical treatments [171], with a change in anisotropy being able to the change the efficacy of hypothermia treatments for example [172]. Superparamagnetic metal nanoparticles have this property (they only present magnetic properties while in the presence of a magnetic field) [173]. However, the benefit of magnetic nanoparticles also presents a potential limitation, due to the toxicity of many magnetic materials [31, 174, 175]. Despite iron being approved for various imaging uses [5, 6, 31], it has been suggested in several studies that naked iron oxide nanoparticles may have some adverse effects when used in cell labeling [176–178]. One method that can be used to overcome any potential toxicity limitations is to coat the iron core [179]. A range of materials can be used as the coating material: silica [180–182], polymers [183, 184], gold [62, 93, 95, 185], or silver [58, 186]. Gold has low pharmaceutical activity [187] and silver has been used in biomedical applications for many years [188, 189],

The combination of a superparamagnetic core with an inert and safe metal coating produces metal nanoparticles with superior characteristics to non-magnetic metal particles [190]. As well as reducing toxicity, the coating also provides the potential for the conjugation of functionalized molecules onto the surface, such as drugs and biomolecules for application in the medical field [97, 140, 152]. It is of note that a core-shell nanoparticle still possesses the properties and uses of a nanoparticle made from the same material as just the shell, but the superparamagnetic core gives the ability to direct the nanoparticle in the body [191]. For example, a gold nanoparticle with an antibody is classified as a targeting nanoparticle, introducing the core would classify the nanoparticle as a directed targeting nanoparticle [173].

## Current Medicinal Uses of Gold-Coated Iron Oxide Nanoparticles

Core-shell superparamagnetic nanoparticles have already been assessed for their biomedical uses, with a wide range of uses already being applied [192] and with a majority of research investigating into the use of gold as a shell for the nanoparticles, in part due to its biocompatibility and ability to easily bind to a variety of materials. As such, this section will deal exclusively with gold shell nanoparticles. One of these uses is as a magnetic carrier for drug targeting [192–196]. Kayal and Ramanujan have tested an in vitro apparatus that simulates the human circulatory system as a test for the magnetic delivery of gold-coated iron oxide nanoparticles (Au-Fe_3_O_4_) loaded with doxorubicin [194]. Their system had various magnetic fields of increasing strength next to a capillary through which the doxorubicin-loaded particles were passed. A significant percentage of these nanoparticles were captured within the magnetic fields, strongly indicating the potential for the use of magnetic nanoparticles in drug delivery. Another use for a targeted system is the application of Au-Fe_3_O_4_ nanoparticles in photothermal therapy. Bhana et al. demonstrated the use of a core-shell system used in combination therapy deployed against two different cancer cell lines; head and neck (KB-3-1) and breast (SK-BR-3) with a reported decrease in cell viability of 64% when they exposed cell lines to a combined photothermal and photodynamic therapy, compared to each modality used on its own [197]. In photothermal therapy, gold nanoparticles are coated with a ligand, such as PEG [142], and these nanoparticles are irradiated with a laser, with a wavelength that matches the UV-vis *λ*-max of the gold nanoparticles [194]. The nanoparticles vibrate at the laser’s frequency which causes heat to be released causing the death of the surrounding tissue [198], introducing a core which is superparamagnetic can allow for a more accurate targeting for use in this therapy. Similarly, it has been reported by Kirui et al. that gold hybrid nanoparticles were deployed against SW1222 colorectal cancer in photothermal therapy, showing an increased case of cellular apoptosis after therapy, with their conclusion being that the cells showed an increased uptake, leading to a reduced laser power required to reach threshold therapeutic levels [199]*.* The use of core-shell nanoparticles for photothermal therapy of cancer has also been reported by other groups [200, 201].

Metal nanoparticles have already shown to have a place in contrast imaging, for example core-shell nanoparticles can also be used in T_1-_ and T_2_-weighted imaging in MRI [202]. Research by Cho et al. demonstrated that gold-coated iron nanoparticles can be successfully used in MRI imaging, as well as opening the route for conjugating various ligands for use in biosensors [202]*.* A magnetic carrier capable of imaging and photothermal therapy has been reported by Cheng et al. They demonstrated the magnetic targeting of multi-functional nanoparticles to a tumor in a mouse model, which could be imaged inside the tumor and showed a reduction in the tumor size when combined with photothermal therapy [203]*.* It is also of note that in this work, both the nanoparticle dosage (1.6 mg/kg) and laser power (1 W/cm^2^) are among the lowest applied for in vivo photothermal therapy. Moreover, there was no obvious toxicity from the nanoparticles reported. Table 6 presents some of the currently reported uses of core-shell nanoparticles.

**Table 6:** Examples of the medical uses already been demonstrated for gold-coated iron magnetic nanoparticles

Type of nanoparticle	Medical application	Ref
Gold-coated iron oxide	Targeted delivery of doxorubicin	[194]
Gold-coated iron oxide	Photothermal and photodynamic combination anticancer treatment	[197]
Gold hybrid nanoparticles	Photothermal anticancer therapy	[199]
Gold-coated iron nanoparticles	T_1_- and T_2_-MRI imaging	[202]
Multifunctional gold nanoparticle	Magnetically directed tumor targeting in mice for phototherapy and imaging of the particles	[203]
Multifunctional gold-coated iron oxide	Cancer diagnosis and therapy	[204]
Gold-coated iron oxide	Cancer therapy	[205]
Gold-coated iron oxide	MRI/PA imaging	[206]

**Fig. 7:**
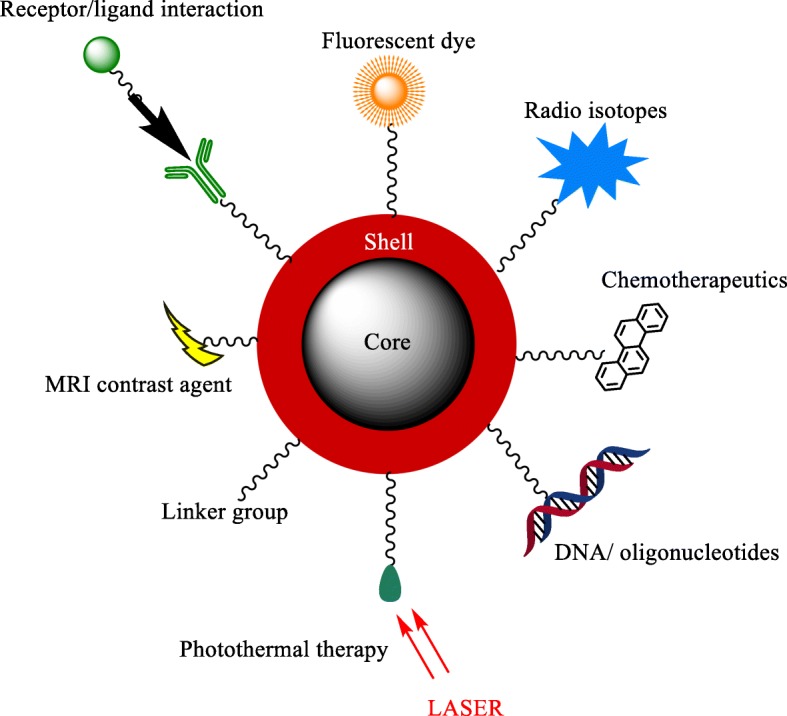
A pictorial representation of the applications of core/shell nanoparticles

Another medical area where such core-shell metal nanoparticles have been suggested to make an impact is in directed enzyme prodrug therapy (DEPT) [170, 191]. DEPT is a promising method of cancer treatment, with several therapies making it through to clinical trials [207, 208]. The main principal of DEPT is the targeted delivery of a prodrug-activating enzyme to a tumor site. Upon arrival at the tumor site, the enzyme enters the target cells where it can later activate an administered prodrug. However, the efficacy of the therapy depends on the ability to direct the enzyme to the tumor site, with current directional techniques relying on passive targeting methods such as viruses [207, 209] or antibodies [210, 211], rather than an active targeting system for enzyme delivery. A novel therapy proposed by Gwenin et al. potentially overcomes the targeting issue [170, 212]. This approach involves conjugating a genetically modified prodrug-activating enzyme onto the surface of a gold-coated iron oxide superparamagnetic nanoparticle (AuMNP), then directing the AuMNP-enzyme conjugate to the target site using a magnetic field to increase the efficacy of the targeted therapy. Figure 7 presents some of the uses of a core-shell nanoparticle.

## Conclusions

In brief, single metal nanoparticles have been shown to currently possess a wide range of biomedical applications, with more application for these nanoparticles being discovered. One of the limiting factors that these nanoparticles face in medical treatments is to find a way for precise accurate targeting of areas within the body, be it for targeting of a drug delivery or for therapies involving the nanoparticles directly. A way to overcome this is to employ a magnetic core to create core-shell nanoparticles that can then be directed around a body using a magnetic field. There are a variety of methods that can be used to synthesize these core-shell nanoparticles, with each method having its own advantages and disadvantages. There remain many obstacles for core-shell nanoparticles before they can be routinely applied in the medical field and these include

1. Achieving a synthesis route which produces easily repeatable results;
2. Producing particles of a set size [22–24] and shape [25–28]; and
3. Producing large enough quantities to make it economically viable [29].

Another key factor is the relatively unknown toxicity of some nanoparticles over an extended period of time due to how relatively new the field of research is.

## Data Availability

Not applicable

## Abbreviations

(o/w): Oil-in-water
(w/o): Water-in-oil
(w/sc-CO_2_): Water-in-supercritical CO_2_
AgNP: Silver nanoparticle
Ag-PS-NPs: Polysaccharide-coated silver nanoparticles
Au-Fe_3_O_4_: Gold-coated iron oxide nanoparticle
Au-MES: Mercaptoethane sulfate-coated gold nanoparticle
AuNP: Gold nanoparticle
Au-PEG-TNF: Polyethylene glycol-coated tumor necrosis factor-loaded gold nanoparticles
CT: Computed tomography
CTAB: Cetyltrimethylammonium bromide
CuI NPs: Copper-iodine nanoparticles
DNA: Deoxyribonucleic acid
FCV: Feline calicivirus
FR: Folate receptor
Gp120: Glycoprotein 120
H1N1: Influenza A of swine origin
HBV: Hepatitis B virus
HIV: Human immune-deficiency virus-1
HSV-1: Herpes simplex virus 1
KB-3-1: Head and neck cancer
MC-38: Colon carcinoma
MPV: Monkeypox virus
MRI: Magnetic resonance imaging
PA: Photoacoustic imaging
PEG: Polyethylene glycol
PVP-AgNP: Polyvinylpyrrolidone-coated silver nanoparticle
RNA: Ribonucleic acid
ROS: Reactive oxygen species
siRNA: Small interfering ribonucleic acid
Sk-BR-3: Breast cancer
SPECT: Single-photon emission computed tomography
TNF: Tumor necrosis factor
